# Clinical application of terminal ileum suspension in laparoscopic radical resection for low rectal cancer

**DOI:** 10.12669/pjms.38.1.4721

**Published:** 2022

**Authors:** Zheng Long-zhi, Zu Bin, Huang Jian-xin, Lin Wei

**Affiliations:** 1 Dr. Zheng Long-zhi, PhD. Associate Chief Physician, Department of Gastrointestinal Surgery, The Affiliated Hospital of Putian University, Putian, Fujian Province, People’s Republic of China; 2 Dr. Zu Bin, Attending Doctor. Department of Gastrointestinal Surgery, The Affiliated Hospital of Putian University, Putian, Fujian Province, People’s Republic of China; 3 Dr. Huang Jian-xin, Attending Doctor, Department of Ultrasonography, The Affiliated Hospital of Putian University, Putian, Fujian Province, People’s Republic of China; 4 Dr. Lin Wei, PhD. Chief Physician, Department of Gastrointestinal Surgery, The Affiliated Hospital of Putian University, Putian, Fujian Province, People’s Republic of China

**Keywords:** Terminal ileum suspension, radical resection,. rectal cancer, Sphincter preservation surgery

## Abstract

**Objectives:**

This paper introduces the surgical procedure of “terminal ileum suspension” in the radical resection for low rectal cancer patients and evaluates the possibility of its clinical application.

**Methods:**

This paper retrospectively analyzed the data of patients with low rectal cancer who underwent “terminal ileum suspension” during radical resection of rectal cancer (Dixon) in our hospital, and introduces the specific surgical procedures and key points of “terminal ileum suspension”. Observe the relevant conditions of patients during the operation, postoperative recovery and postoperative complications, and analyze the safety and feasibility of this operation (“terminal ileum suspension”).

**Results:**

The operation of all 8 patients went smoothly, and no anastomotic leakage, intestinal obstruction, and open diversion of suspended terminal ileum were found. The application of “terminal ileum suspension” in the operation of low rectal cancer has achieved ideal clinical effect, without increasing the rate of anastomotic leakage and rehospitalization, reducing the proportion of the secondary return operation, and reducing the pain of the patients.

**Conclusion:**

“Terminal ileum suspension” is a safe, effective and feasible surgical method for laparoscopic radical resection of low rectal cancer, which can be applied in clinical practice.

## INTRODUCTION

Rectal cancer is one of the most common malignant tumors of the digestive system, and its incidence has been increasing year by year in the past 20 years, among which the low rectal cancer accounts for 70% ~ 80% of all rectal cancers.^1^ With the introduction of total mesorectal resection (TME), the development of laparoscopic technology and the upgrading of medical equipment, patients with low/ultra-low rectal cancer have the opportunity to preserve the anus, but the probability of anastomotic leakage after preserving the anus is also significantly increased. It has been reported in the literature that the incidence of anastomotic leakage after rectal cancer surgery is 1% ~ 21%, and the common reports are between 5% ~ 10%.^2^

Anastomotic leakage is the most serious postoperative complication in patients with low/ultra-low rectal cancer. It not only affects the postoperative rehabilitation of patients, but even endangers their lives in serious cases. Therefore, how to prevent or reduce postoperative anastomotic leakage and its complications has always been the focus of surgeons’ research. At present, the prophylactic stoma is believed to reduce the adverse consequences of anastomotic leakage after sphincter-preserving surgery for low rectal cancer, and can reduce the incidence of anastomotic leakage for reoperation, but the postoperative stoma and the secondary reurn operation brought great inconvenience and psychological pressure to the patients, and seriously affected the quality of life of the patients. Therefore, how to predict the low-risk population of anastomotic leakage more accurately, improve anastomosis and drainage, and reduce the prophylactic stoma of the low-risk population are also the future efforts of colorectal surgeons.

According to the previous clinical experience of our departments and the surgical experience of other experts,^3^ we proposed the surgical method of “terminal ileum suspension” in the radical operation of patients with low/ultra-low rectal cancer. This operation is concise and practical, and the operation is simple for the secondary return of intestines, it avoids the pain of prophylactic stoma for patients without anastomotic leakage. Here we introduce in detail a new prophylactic stoma surgery method - “terminal ileum suspension”, and summarize the safety and feasibility of this operation method.

## METHODS

This study was an observational study conducted in our hospital from January 2020 to June 2020. The study included patients undergoing radical resection of low rectal cancer (Dixon) with terminal ileum suspension during this period, and a total of 8 patients were evaluated during this period.

We selected patients: selected patients with low rectal cancer admitted to our hospital from January 2020 to June 2020. The clinical stage of preoperative MRI evaluation was stage I-III a, and eight cases were feasible for radical resection. The male to female ratio was 5:3, the median age was (62.8 ± 9.6) years, the distance from the lower margin of the tumor to the anal margin was (6.63 ± 1.78) cm, and the body mass index (BMI) was (22.3 ± 2.5) kg/m^2^.

### Inclusion Criteria:

• Patients with low rectal cancer diagnosed clearly by colonoscopy and pathological examination and with the lower margin of the tumor 5 ~ 8cm from the anal margin;
• Chest and abdomen CT/MRI examination of patients without distant metastasis of liver, lung, etc., the estimated survival time is ≥3 months;
• Standardized laparoscopic-assisted radical resection of low rectal cancer (Dixon) was performed.

### Exclusion criteria:

• Patients with severe organ dysfunction;
• Patients with tumor complicated with hemorrhage, perforation or obstruction requiring emergency surgery;
• Patients requiring combined organ resection.
• Patients and their families were fully informed and informed consent was obtained before the operation, and the hospital’s ethics committee approved the operation.

### Surgical technique:

**Fig.1:**
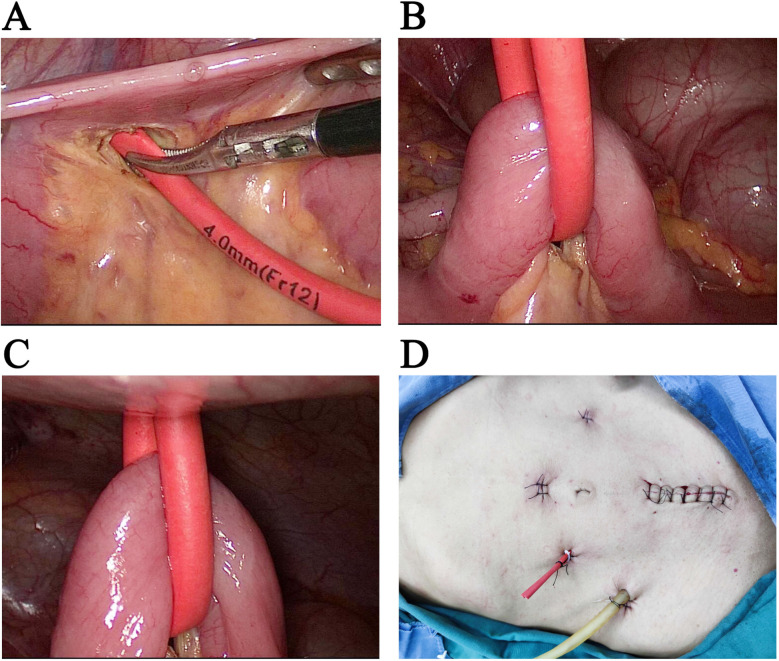
**A,** The mesangium of the terminal ileum is separated, and a no. 12 urinary catheter is placed in preparation for traction of the intestine. **B,** The suspended terminal ileum must be free from mesenteric tension and hanging freely in the abdominal cavity to prevent intestinal obstruction. **C,** There is a 2-3mm gap between the suspended terminal ileum and the abdominal wall to prevent obstruction of the small intestine and discomfort caused by peristalsis. At the same time, attention should be paid to avoid the possibility of internal abdominal hernia caused by a large gap. **D,** The urinary catheter for traction of the terminal ileum is drawn from the auxiliary trocar (5mm trocar) in the right lower abdomen and secured to the skin with a silk thread.

All the included patients underwent laparoscopic radical resection of low rectal cancer (Dixon) in accordance with the principles of TME. After the colon-anal anastomosis was completed, the terminal ileum was searched with ileocecal junction as the marker. At the mesentery of small intestine about 15-20 cm away from the ileocecal junction, separate the mesentery of small intestine with an electrocantery or ultrasound scalpel, and insert a no. 12 red urinary catheter (Fig-1). The intestines hung freely on the abdominal wall, making the intestines gently close to the abdominal wall to prevent postoperative mechanical small intestinal obstruction and discomfort caused by intestinal peristalsis (Fig-2,3). The urinary catheter was pulled out of the body from the auxiliary trocar (5mm trocar) on the right side of the patient, and the intestines remain in the abdominal cavity without having to be pulled out of the abdominal wall. After the urinary catheter was drawn out of the body, the urinary catheter was fixed near the abdominal wall surface with a harmlock clip, and the urinary catheter was sutured to the skin with a no. 4 silk(Fig-4). A double cannula drainage was routinely placed near the anastomosis of the pelvic floor for drainage.

If the patient recovers well after the operation and the anastomotic leakage is clinically excluded, then the pelvic double cannula drainage and the terminal ileum preset urinary catheter can be removed 10-12 days after the operation. *Indication of open preset intestine*: anastomotic leakage rating of B or above. In the case of general anesthesia/local anesthesia, the incision at the preset intestine was expanded, the intestine was pulled out, and a 25 mm circular stapler for single-layer anastomosis with skin to complete the stoma, so as to reduce the related complications caused by anastomotic leakage and promote the healing of anastomotic leakage.

## RESULTS

As shown in Table-I, the eightpatients undergoing “Terminal Ileum Suspension” included five men (62.5%) and three women (37.5%) with a mean age of 62.8 ± 9.6 years (range, 35-75 years) and a mean body mass index (BMI) of 22.3 ± 2.5 kg/m^2^ (range, 18.4-28.6 kg/m^2^). The average distance from the lower margin of the tumor to the anal margin was 6.63 ± 1.78 cm. The postoperative pathological TNM stage included Stage-II (n=2, 25%), Stage-III (n=6, 75%).

**Table-I:** General information of the patients enrolled in "Terminal Ileum Suspension".

Variable	Value
***Gender, n (%)***	
Male	5 (62.5)
Female	3 (37.5)
Age [years]	62.8 ±9.6
BMI [kg/m^2^]	22.3 ±2.5
*The average distance from the lower margin of the tumor to the anal margin [cm]*	6.63 ±1.78
***TNM stage***	
Stage-II	2 (25)
Stage-III	6 (75)

As shown in Table-II, the operations of the eight patients were successfully completed. The mean operation time was 208.3 ± 20.5 min (range, 155-210 minutes),and the mean estimated blood loss was 48.4 ± 10.8 ml. The mean times to the first anal exhaust time and the time of off-bed activity were 72.8 ± 6.2 h and 18.6 ± 3.7 h, respectively; and the mean postoperative hospital stay was 11.7 ± 2.1 d. All the patients in the group successfully completed Dixon surgery and “Terminal Ileum Suspension”, achieving anal preservation. The postoperative complications of the patients undergoing “Terminal Ileum Suspension” included one case of anastomotic bleeding, two cases of lung infection, and two cases of urinary retention, all of which were successfully treated conservatively. The patients were discharged successfully and no recurrence or metastasis was found after a median follow-up period of six months (range, 3-9 months).

**Table-II:** Outcomes of patients undergoing "Terminal Ileum Suspension".

Variable	Value
Operation time [min]	208.3 ±20.5
Blood loss [ml]	48.4 ±10.8
First anal exhaust time (h)	72.8 ±6.2
*The time of off-bed activity (h)*	18.6 ±3.7
Postoperative hospital stay (d)	11.7 ±2.1
The number of cases with preset ileum undergoing ileostomy, n (%)	0
***Postoperative complications, n***	
Anastomotic leakage	0
Anastomotic hemorrhage	1
Lung infection	2
Incision infection	0
Urinary retention	2

## DISCUSSION

Clinically, rectal cancer with a lower margin of tumor 5 ~ 8 cm from the anal margin is usually called low rectal cancer, while rectal cancer with a margin of less than five cm from the anal margin is called ultra-low rectal cancer.^4^ Since 1908, Miles first proposed abdominoperineal resection (APR), APR has quickly become the standard surgical procedure for low and ultra-low rectal cancer.^5^ According to Miles, there is no possibility of sphincter preservation in low or ultra-low rectal cancer; however, with the in-depth study of rectal cancer biology, more and more researchers have found that low and ultra-low rectal cancer still have the possibility of sphincter preservation, and the improvement of equipment and the development of surgical techniques provide more opportunities for this type of rectal cancer patients to achieve sphincter preservation.

However, the incidence of anastomotic leakage after sphincter preservation surgery was also significantly increased in patients with low/ultra-low rectal cance.^6^ Anastomotic leakage (AL) is a common serious complication of low rectal cancer surgery, literature reported that the incidence of anastomotic leakage after rectal cancer between 2.4% ~ 15.9%, and the fatality rate after anastomotic leakage can be as high as 16%.^2^ Anastomotic leakage not only affects the postoperative rehabilitation of patients, increases the length of hospitalization and the cost of hospitalization, but also delays the opportunity of postoperative adjuvant therapy, such as radiotherapy and chemotherapy. In serious cases, it even endangers the lives of patients, may increase the fatality rate and local recurrence rate of tumors, and lead to the reduction of long-term survival rate of patients.^7^,^8^

Prophylactic stoma can minimize the stimulation of stool to the anastomotic stoma, reduce the serious adverse reactions after anastomotic leakage in low rectal cancer, reduce the rate of secondary operation, and facilitate the patients to recover more quickly. Therefore, it is recommended to perform a temporary prophylactic stoma for rectal cancer patients undergoing low anterior rectal excision.^9^,^10^ But the reverse side, compared with patients without prophylactic stoma, prophylactic stoma increased the overall short-term postoperative complication rate, especially the stoma-related complications can affect the quality of life,^11^ and even some prophylactic stoma will eventually become permanent stoma for many reasons. In addition, the secondary operation of prophylactic stoma may cause complications such as incision infection, anastomotic fistula, intestinal obstruction, and even death.^12^,^13^

For this reason, many researchers have invented some improved surgical methods, such as unopened loop ileostomy, non-return ileostomy and terminal ileal external placement in order to give consideration to both the retention of anus and postoperative life quality of patients with low rectal cancer.^14^,^15^,^16^ Although these surgical methods, to a certain extent, avoid the secondary operation when anastomotic leakage occurs and the surgical trauma brought to the patients when the stoma is retracted, they have certain advantages. However, the changes of intestinal environment caused by foreign bodies in the intestine and the external placement of the intestine may lead to psychological and physiological trauma of the patients after the operation. Based on this, “terminal ileum suspension” is adopted in radical resection of low/ultra-low rectal cancer patients, which can reduce related complications caused by the reoperation of anastomotic leakage. The operation is simple when the intestine is returned, and the pain of ileostomy is avoided in patients without anastomotic leakage. When anastomotic leakage occurs, patients undergoing terminal ileostomy by suspension do not increase the difficulty of ileostomy. At the same time, for patients who are older and have poor general conditions and cannot tolerate secondary operations under general anesthesia , the ileostomy can be completed under local anesthesia, which can effectively reduce the risk of anesthesia, and the operation is simple and practical.

The technique of this operation is to leave the terminal ileum intended for stoma in the abdominal cavity without pulling it out. A urinary catheter is used to pass through the terminal ileal mesentery to suspend the intestines, so that the terminal ileum is easily drawn to the abdominal wall. The urinary catheters is drawn out of the abdominal cavity and fixed to the skin, which can reduce the influence of the external environment on the intestine and reduce the occurrence of inflammation and infection in the intestinal wall. When the postoperative recovery of patients with low rectal cancer is good and there is no anastomotic leakage clinically, the incision on the abdominal wall can be closed by removing the urinary catheter on the 10th to 12th day after the surgery, thus avoiding the secondary operation of returning the ostomy intestines. In case of anastomotic leakage, the incision at the preset urinary catheter can be enlarged under local anesthesia or general anesthesia, and the urinary catheter can be used as a guide to pull the terminal ileum suspended in the abdominal wall out of the abdominal cavity, and the ileostomy can be completed under direct vision. The operation is simple and effective, and the wound is small.

Of course, the key to prevent anastomotic leakage lies in the satisfactory colon-anal anastomosis technology, good blood circulation at the anastomotic site and no tension. The intestine freely drops on the pelvic surface of sacrum and cannot arch up like an arch bridge. If necessary, the splenic flexure of the colon can be further dissociated to ensure no tension at the anastomotic site. In addition, due to the formation of two intersecting angles on the side of the anastomotic (“dog’s ear area”), the staples here cross each other and have a weak structure. We usually use proline sutures to reinforce the anastomosis- dog’s ear area to reduce risk of anastomotic leakage.^17^-^19^ Our experience with this surgical method-”terminal ileum suspension” is: for the suspended terminal ileum, it should be arranged laterally and laterally as much as possible under laparoscopy, and the intestinal mesangium should not have tension. A 2-3mm gap can be retained between the suspended ileum and the abdominal wall to prevent postoperative small intestinal obstruction and discomfort caused by peristalsis. Of course, it is also necessary to avoid the possibility of internal hernia caused by the large gap between the terminally suspended ileum and the abdominal wall.

### Limitations of the study:

It includes too small sample size

## CONCLUSION

Our preliminary results show that the application of “terminal ileum suspension” in laparoscopic radical resection of low rectal cancer is a safe and feasible surgical method, especially for patients with low rectal cancer undergoing sphincter preservation surgery. When doctors are hesitating whether to perform prophylactic stoma, “terminal ileum suspension” can yet be regarded as a kind of ideal choice. At present, the number of cases of selecting “terminal ileum suspension” in laparoscopic radical resection of low rectal cancer is still relatively small, and the incidence of postoperative anastomotic leakage and the complications caused by the operation itself have not been sufficiently studied, so more clinical studies are needed to demonstrate. Surgical techniques also need to be perfected in clinical practice by surgeons.

### Authors’ Contribution:

**ZLZ:** Study concept and editing of manuscript.

**LHB & HJC:** Literature search**,** Data collection and statistical analysis.

**LW:** Corresponding author, the consultant surgeon who operated on the cases..
